# Establishment and validation of a polygene prognostic model for clear cell renal cell carcinoma

**DOI:** 10.3389/fgene.2022.1021163

**Published:** 2022-10-20

**Authors:** Kai Gan, Keying Zhang, Yu Li, Xiaolong Zhao, Hongji Li, Chao Xu, Shaojie Liu, Chao Zhang, Donghui Han, Weihong Wen, Weijun Qin

**Affiliations:** ^1^ Department of Urology, Xijing Hospital, Fourth Military Medical University, Xi’an, China; ^2^ Department of Medical Research, Northwestern Polytechnical University, Xi’an, China

**Keywords:** ccRCC, prognostic, bioinformatics, tumor-associated pathways, polygene

## Abstract

**Purpose:** To establish an effective prognostic model for patients with clear cell renal cell carcinoma (ccRCC).

**Methods:** We identified four hub differentially expressed genes (DEGs) in Gene Expression Omnibus (GEO) database and verified them in the Cancer Gene Atlas (TCGA), STRING, UALCAN, TIMER, and Gene Expression Profiling Interactive Analysis (GEPIA) databases. We then used TCGA and International Cancer Genome Consortium (ICGC) to identify tumor pathway molecules highly correlated with hub DEGs. And by further LASSO and Cox regression analysis, we successfully identified five genes as prognostic factors.

**Results:** We successfully identified a risk prediction model consisting of five genes: IGF2BP3, CDKN1A, GSDMB, FABP5, RBMX. We next distributed patients into low-risk and high-risk groups using the median as a cutoff. The low-risk group obviously had better survival than those in the predicted high-risk group. The results showed discrepancies in tumor-associated immune cell infiltration between risk groups. We also combined the risk model with clinical variables to create a nomogram.

**Conclusion:** Our model has a satisfactory predictive effect on the prognosis of ccRCC patients and may provide new ideas for future immune therapy.

## Introduction

According to the latest international epidemiological studies, renal carcinoma causes approximately more than 180,000 deaths each year globally (Sung et al., 2021). The most common subtype of kidney cancer is clear cell renal cell carcinoma (ccRCC), accounting for about three-quarters of all kidney cancer cases (Moch et al., 2016). Although localized ccRCC can be treated with surgical or ablation interventions, some patients may still experience disease recurrence after treatment. Even among patients with early-stage ccRCC, 30% of them will develop tumor progression and metastasis after surgical resection (Lam et al., 2006; Jonasch et al., 2014). Postoperative progression of ccRCC will induce patients into the late phase of tumorigenesis, which is associated with shorter survival (Bedke et al., 2017).

In addition to traditional surgery, radiotherapy and chemotherapy, molecular targeting has also been widely used in the treatment of ccRCC (Brugarolas, 2014). In 2016, Food and Drug Administration (FDA) approved the clinical application of cabozantinib, which is a kind of small molecule tyrosine kinase inhibitor (TKI) (Choueiri et al., 2016). For patients with advanced metastatic ccRCC, the programmed death 1 (PD-1) checkpoint inhibitor nivolumab has been reported to improve overall survival to some extent (Motzer et al., 2015). Humanized programmed death-ligand 1 (PD-L1) related drug atezolizumab can also play a certain anti-tumor role according to recent reports (McDermott et al., 2016). However, the effectiveness of molecular-guided targeted therapy for ccRCC varies greatly from person to person. Not all patients can benefit from treatment. Thus, there is an urgent requirement for us to determine new therapeutic and predictive markers.

Many researchers have used bioinformatics techniques to assess the correlation between gene expression and the progression of cancer (Huang et al., 2021; Zhang et al., 2021). For ccRCC, not many genes have been shown to have prognostic significance. Prognostic models based on coding genes are also lacking. New disease risk prediction models are urgently needed.

**FIGURE 1:**
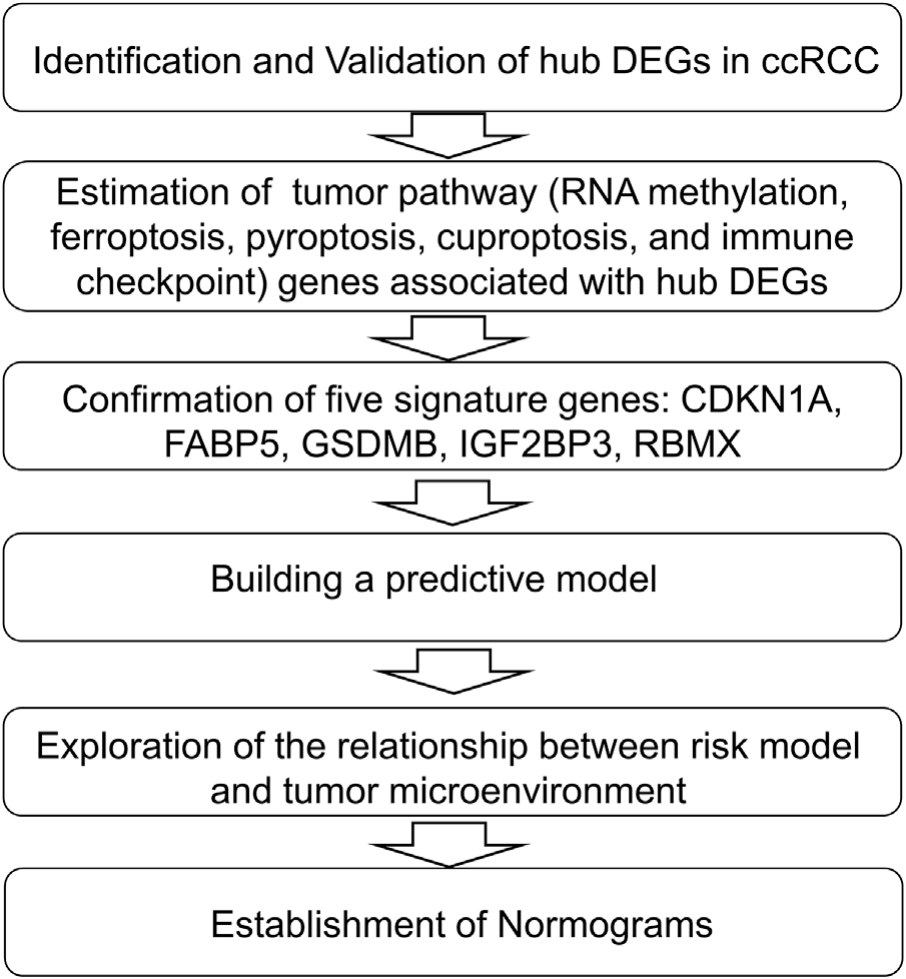
The flow chart of our study.

In this study, we performed an in-depth bioinformatics analysis of hub differentially expressed genes (DEGs) and multiple related molecular pathways for ccRCC using public databases. Finally, we constructed a prognostic model and nomogram. The flow chart of our study was shown in Figure 1.

## Materials and methods

### Primitive data collection

The Gene expression original data along with relevant clinical information can be obtained from Gene Expression Omnibus (GEO) (https://www.ncbi.nlm.nih.gov/geo/) and the Cancer Gene Atlas (TCGA) database (https://portal website.gdc.cancer.gov/). We downloaded four microarray expression profiling datasets from the GEO database: GSE12606, GSE15641, GSE72304, and GSE105261. The latest RNA-Seq data and clinical follow-up information were from the TCGA-KIRC cohort, and gene expression levels were Log2 (TPM + 1).

### Related differentially expressed genes screening

Gene sequencing information extracted from the GEO database was used for comparing differential gene expression between ccRCC and normal kidney tissue through the DESeq method. We concluded that genes with a false discovery rate (FDR) adjusted *p*-value < 0.05 and an absolute value of log2 (fold change) > 1 were considered statistically significant. Volcano plots were generated using the ggplot2 package in R. We analyzed survival data using COX regression and log-rank methods. Kaplan-Meier curves and forest plot figures were drawn using the survminer R package.

### STRING database

We retrieved protein–protein interaction data from the STRING database (https://string-db.org/). Experimentally-determined interaction and text-mining data were considered. The detailed introduction to this database can be found in the article by Szklarczyk et al. (2019).

### UALCAN website

Quantitative analysis of protein was conducted using online National Cancer Institute’s Clinical Proteomic Tumor Analysis Consortium (CPTAC) proteomic results, which were obtained from the UALCAN website. Information about this website can be found in previous literature (Uhlen et al., 2010; Uhlén et al., 2015; Chandrashekar et al., 2017; Thul et al., 2017).

### TIMER database

The TIMER 2.0 database (https://cistrome.shinyapps.io/timer/) was used for gene expression differential analysis. Boxplots were used to show the distribution of gene expression levels and the Wilcoxon test was performed to assess whether the expression difference was statistically significant.

### Gene expression profiling interactive analysis

Gene-expression datasets were analyzed by using the GEPIA tool (http://gepia.cancer-pku.cn/index.html). Center lines in boxplots indicated the median, the boundaries of the box represented 25%–75%, and the whiskers showed 5%–95%. Scatter plots represented the correlation between our riskscore and gene expression level based on the Spearman coefficient.

### International cancer genome consortium

Part of the RNA-sequencing expression profiles were downloaded from ICGC (https://dcc.icgc.org/releases/current/Projects). Gene correlation plots were plotted by the R software package ggstatsplot. *p* < 0.05 was considered statistically significant.

### Establishment of a prognostic model

When building the risk prediction model, LASSO regression analysis was performed to single out genes highly relevant with prognosis. Then, multivariate Cox regression analysis was done to evaluate the initially screened genes. Finally, five genes were successfully incorporated into the ccRCC prognostic model. The risk score was calculated by the following formula: $RiskScore=\overset{n}{\underset{i=1}{\sum }}\left(coefi\times Expi\right)$ .

### Quantification of immune infiltration

The gene set variation analysis (GSVA) was used to calculate individual immune cell infiltration in the TCGA cohort, involving 24 different immune cell types. Overall immune infiltration was carried out by the single sample gene set enrichment analysis (ssGSEA) and ESTIMATE algorithm based on the Spearman coefficient.

### Statistical analysis

In processing the data, we used SPSS 25.0 (IBM Corp, Armonk, NY) and R version 4.1.2 software for statistical analysis. All *p*-values were the results of two-sided tests, and *p* < 0.05 was considered significantly different.

## Results

### Identification of hub DEGs in ccRCC

**FIGURE 2:**
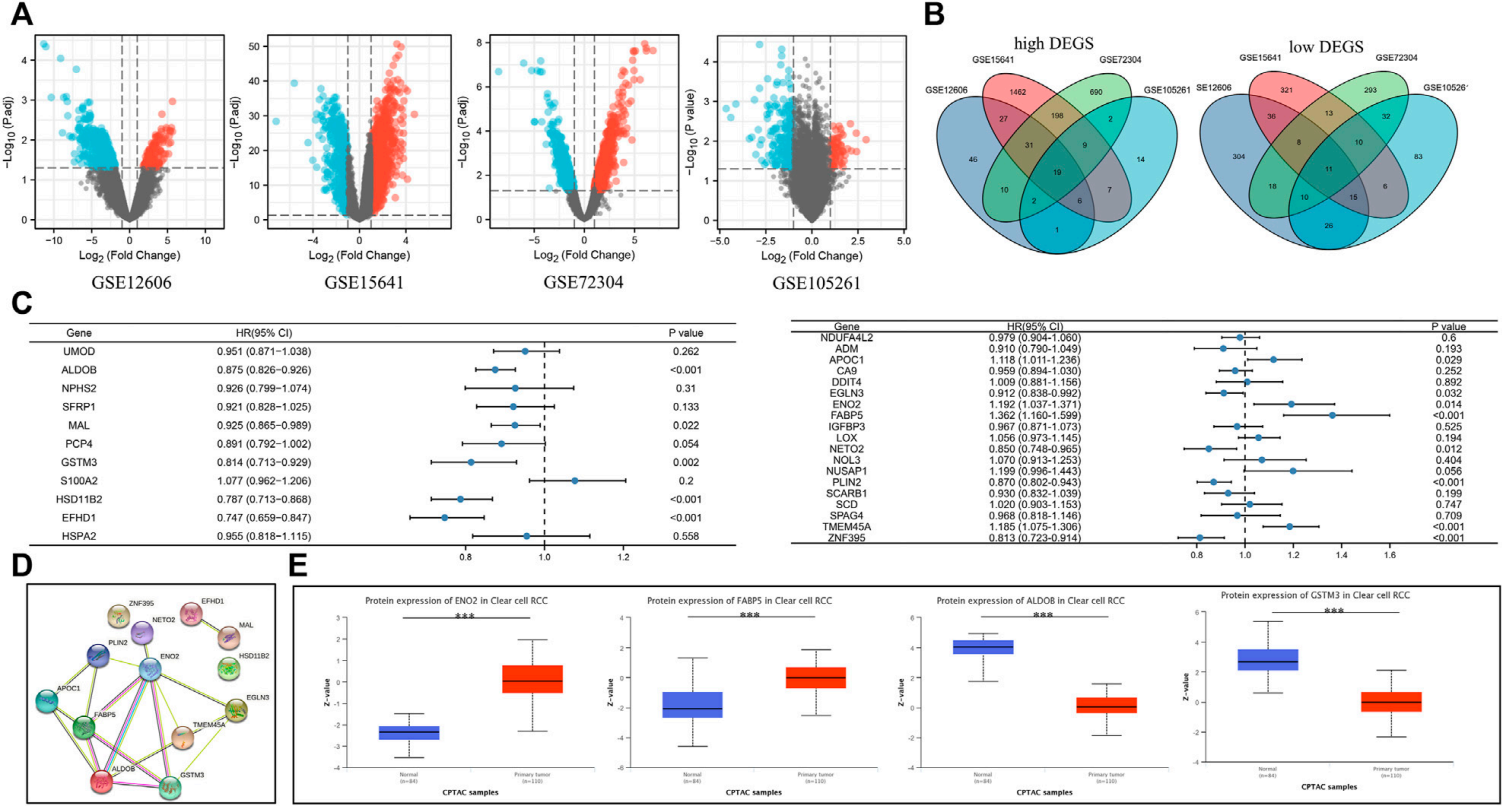
The DEGs in ccRCC. **(A)** Volcano plots showing the gene sequencing information extracted from four microarray expression profiling datasets: GSE12606, GSE15641, GSE72304, and GSE105261. Red indicates upregulated genes and blue indicates down-regulated genes. **(B)** Venn diagram showing the number of common DEGs in the four sequencing files. **(C)** Forest plot showing the relationship between common DEGs and overall survival of ccRCC. **(D)** The PPI network analysis of common DEGs using the STRING tool. **(E)** Quantitative analysis of protein levels of four hub DEGs using the online CPTAC proteomic data. DEGs, differentially expressed genes; ccRCC, clear cell renal cell carcinoma; PPI, protein-protein interaction; CPTAC, National Cancer Institute’s Clinical Proteomic Tumor Analysis Consortium; HR, hazard ratio; CI, confidence interval; ***, *p* < 0.001.

**FIGURE 3:**
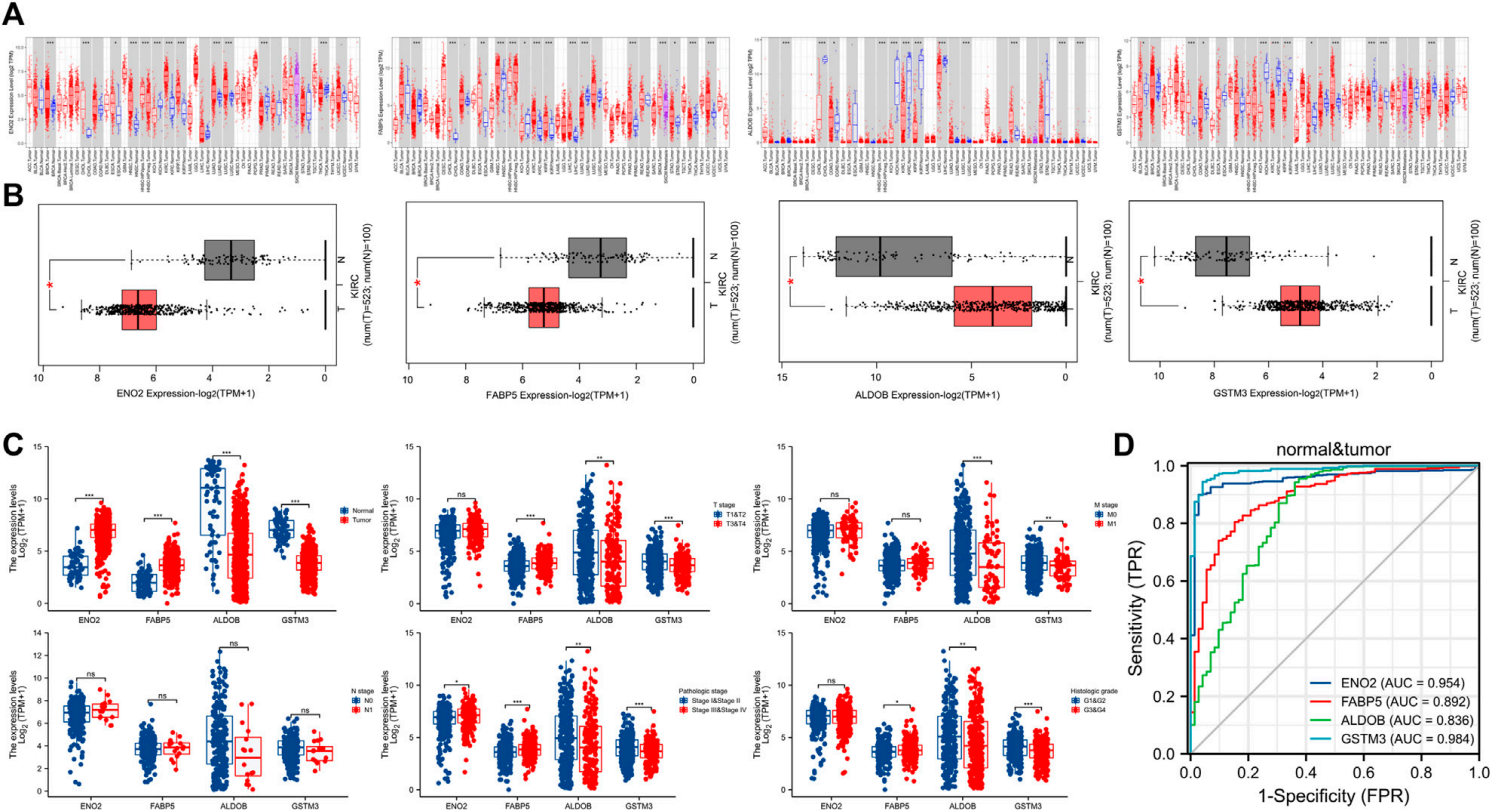
Validation of hub DEGs. **(A)** Exploring the expression of hub DEGs in multiple types of tumors using the TIMER database. **(B)** Exploring the expression of hub DEGs between KIRC (T) and adjust normal tissues (N) using GEPIA database. **(C)** Correlation between hub DEGs and tumor status (clinical stage, pathologic stage, and histologic grade). **(D)** ROC curve of the hub DEGs. DEGs, differentially expressed genes; KIRC, Kidney renal clear cell carcinoma; ROC, Receiver operating characteristic; ns, *p* ≥ 0.05; *, *p* < 0.05; **, *p* < 0.01; ***, *p* < 0.001.

In our research, 52 localized ccRCC tissues and 40 adjust normal tissues from GSE12606, GSE1564, GSE72304, and GSE105261 were included in the analysis. The DEGs of these four gene expression profiles were visualized using volcano plots (Figure 2A). For details of raw gene expression analysis, please see Supplementary Table S1. The sample normalized box, principal component analysis (PCA), and uniform manifold approximation and projection (UMAP) plots can be found in Supplementary Figure S1. Venn diagram analysis identified 19 upregulated genes and 11 downregulated genes among these four GSE datasets (Figure 2B). Subsequently, a univariate Cox regression survival analysis of DEGs was performed based on the TCGA database (Figure 2C). After the above analysis, we concluded that the prognostic related DEGs were: ALDOB, APOC1, EFHD1, EGLN3, ENO2, FABP5, GSTM3, HSD11B2, MAL, NETO2, PLIN2, TMEM45A, ZNF395. We then performed protein-protein interaction (PPI) network analysis using the STRING tool in these genes. Proteins with an interaction score >0.150 and *p* < 0.05 were displayed in Figure 2D. We screened out four hub DEGs: ENO2, FABP5, ALDOB, GSTM3. The specific correlation coefficient of these four genes can be found in Supplementary Table S2. Quantitative analysis of relative protein expression level showed that two genes (ENO2, FABP5) were upregulated and two genes (ALDOB, GSTM3) were down-regulated in ccRCC compared to normal samples (Figure 2E). Gene level expression analysis results in TIMER (Figure 3A) and GEPIA (Figure 3B) were consistent with protein level analysis. We also examined the association of hub DEGs with clinical tumor grades in the TCGA database (Figure 3C). Combined with the Receiver operating characteristic (ROC) curve (Figure 3D), we demonstrated that hub DEGs were associated with the tumor stage.

### Construction of a predictive model based on RNA methylation (m6A), ferroptosis, pyroptosis, cuproptosis, and immune check-point patheway genes

**FIGURE 4:**
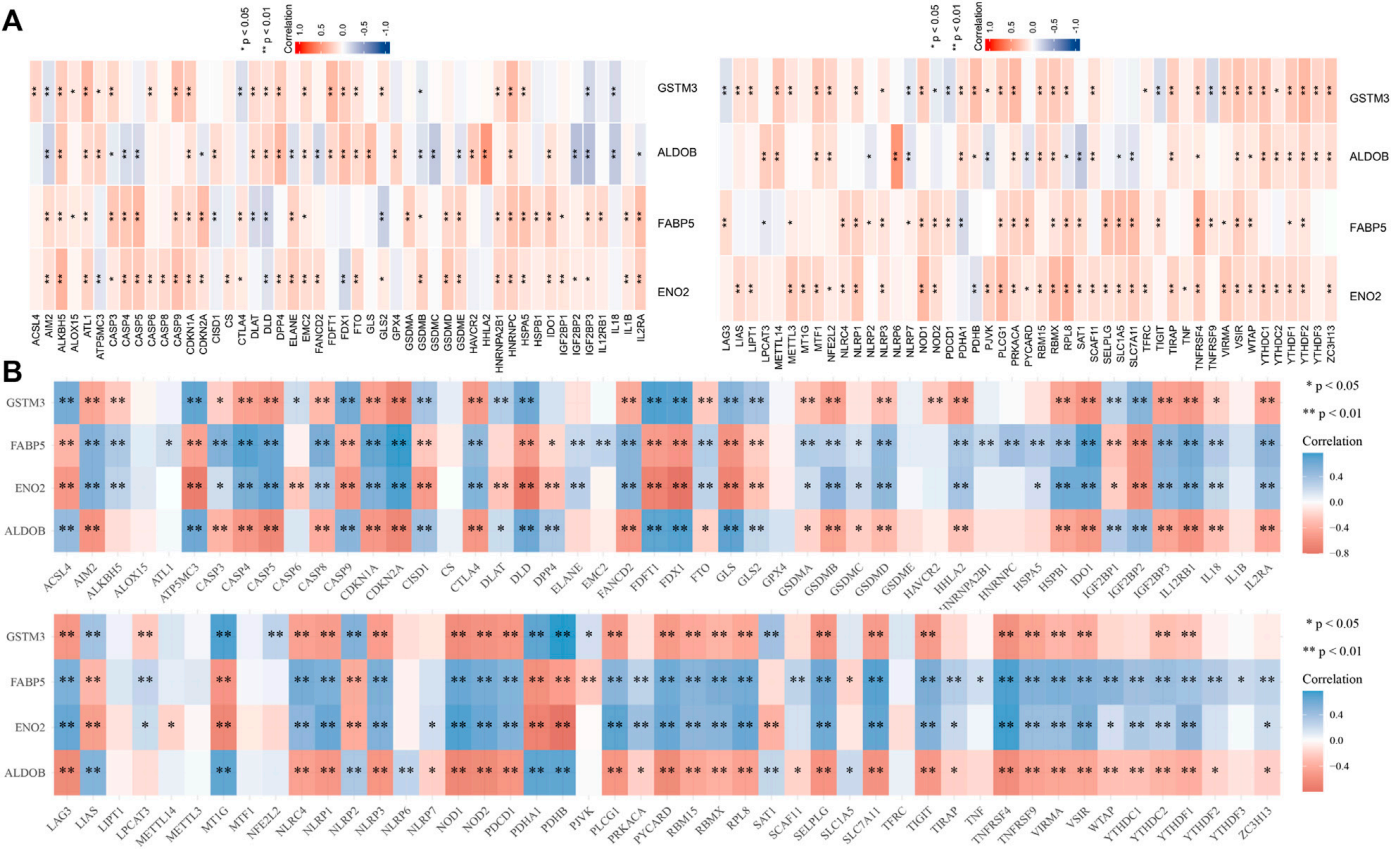
Heatmap showing tumor pathway (RNA methylation, ferroptosis, pyroptosis, cuproptosis, and immune checkpoint) genes associated with hub DEGs in TCGA **(A)** and ICGC **(B)** database. DEGs, differentially expressed genes; TCGA, the Cancer Gene Atlas; ICGC, International Cancer Genome Consortium; *, *p* < 0.05; **, *p* < 0.01.

To analyze the relationship between hub DEGs and tumor signaling pathways in ccRCC, we performed a series of correlation sensitivity analyses. We used heatmaps to reveal the association between hub DEGs and key genes (Supplementary Table S3) in m6A (Li et al., 2019; Zhang et al., 2020a; Yi et al., 2020), ferroptosis (Liu et al., 2020; Chen et al., 2021), pyroptosis (Tang et al., 2020; Hou et al., 2021; Tan et al., 2021; Wang et al., 2021), cuproptosis (Tsvetkov et al., 2022), and immune checkpoint (Braun et al., 2021) pathways by exploring TCGA (Figure 4A) and ICGC database (Figure 4B). Spearman correlation coefficient (r) and *p*-values were displayed in Supplementary Table S4. *p*-value smaller than 0.05 was considered significant. As a result, we found that AIM2, CASP3, CDKN1A, DLD, GSDMB, IGF2BP3, RBMX, RPL8, VSIR, and YTHDF1 were correlated with hub DEGs.

### Risk model building

**FIGURE 5:**
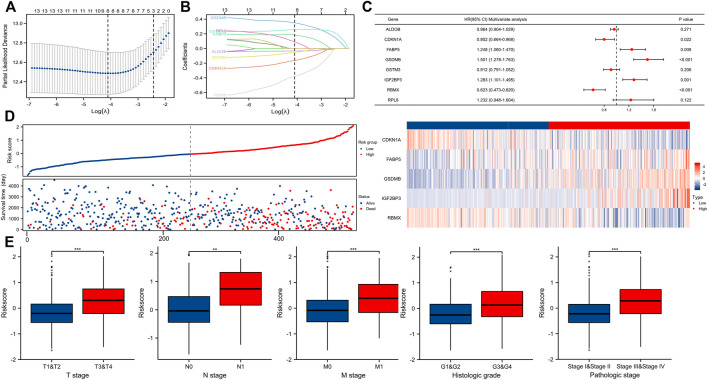
Building a predictive model. **(A,B)** Determination of the number of factors by the LASSO analysis. **(C)** Forest plot showing multivariate COX analysis results of the genes screened in Figures 5A,B. **(D)** Risk factor map for survival. **(E)** Correlation between riskScore and tumor stage (T, N, M, pathologic stage, and histologic grade).

**FIGURE 6:**
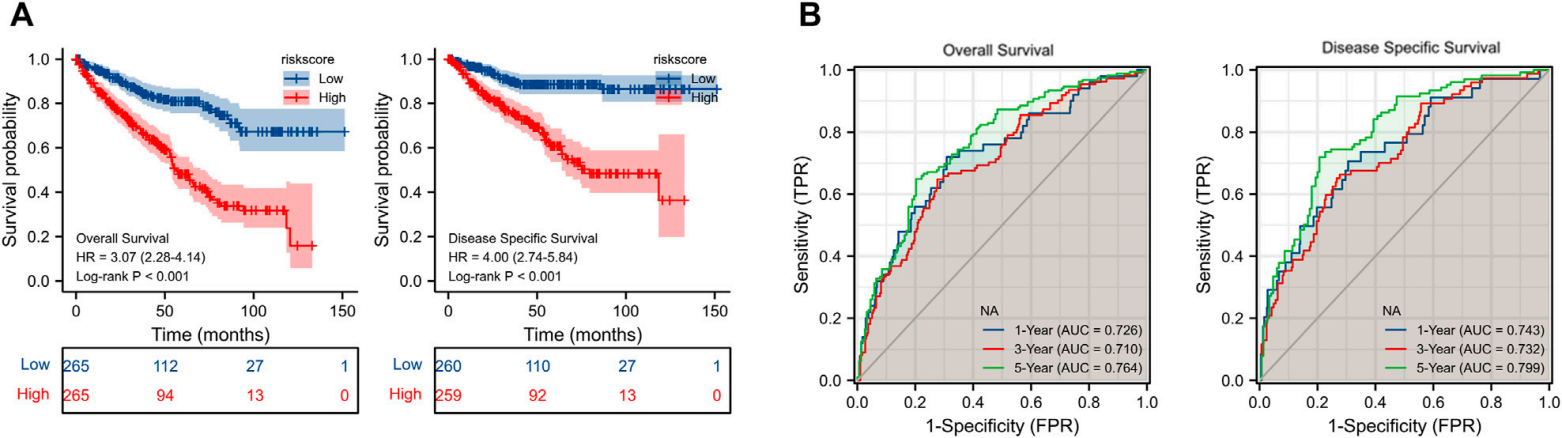
**(A)** KM survival curve for OS and DSS of our model in TCGA cohort. **(B)** ROC curve of prognosis model for OS and DSS. KM, Kaplan-Meier; OS, overall survival; DSS, disease-specific survival; TCGA, the Cancer Gene Atlas; ROC, Receiver operating characteristic.

By using LASSO (Figures 5A,B) together with multivariate Cox regression (Figure 5C) to analyze hub DEGs (ENO2, FABP5, ALDOB, GSTM3) and related pathway genes (AIM2, CASP3, CDKN1A, DLD, GSDMB, IGF2BP3, RBMX, RPL8, VSIR, and YTHDF1), we finally selected five signature genes (CDKN1A, FABP5, GSDMB, IGF2BP3, RBMX) in total to construct the prognostic model (Figure 5D). This model was established to evaluate the survival risk for each TCGA sample as follows: RiskScore = 2.953 + IGF2BP3 × 0.317 + CDKN1A × −0.221 + GSDMB×0.398 + FABP5 × 0.260 + RBMX × −0.501. The risk value, survival time, and gene expression parameter for each TCGA sample can be found in Supplementary Table S5. We used the median as our population segmentation value and then divided the TCGA ccRCC patient cohort into low-risk and high-risk groups. The tumor stage in high-risk group was significantly higher than that in low-risk group (Figure 5E). The high-risk group had obviously worse overall survival (OS) and disease-specific survival (DSS) than the low-risk group (Figure 6A). The area under the curve (AUC) values of risk score for 1-, 3-, and 5-year OS were 0.726, 0.710, and 0.764, respectively. And for 1-, 3-, and 5-year DSS, AUC values of risk score were 0.743, 0.732, and 0.799 (Figure 6B).

### Relationship between our risk model and immune invasion in ccRCC tumor microenvironment

**FIGURE 7:**
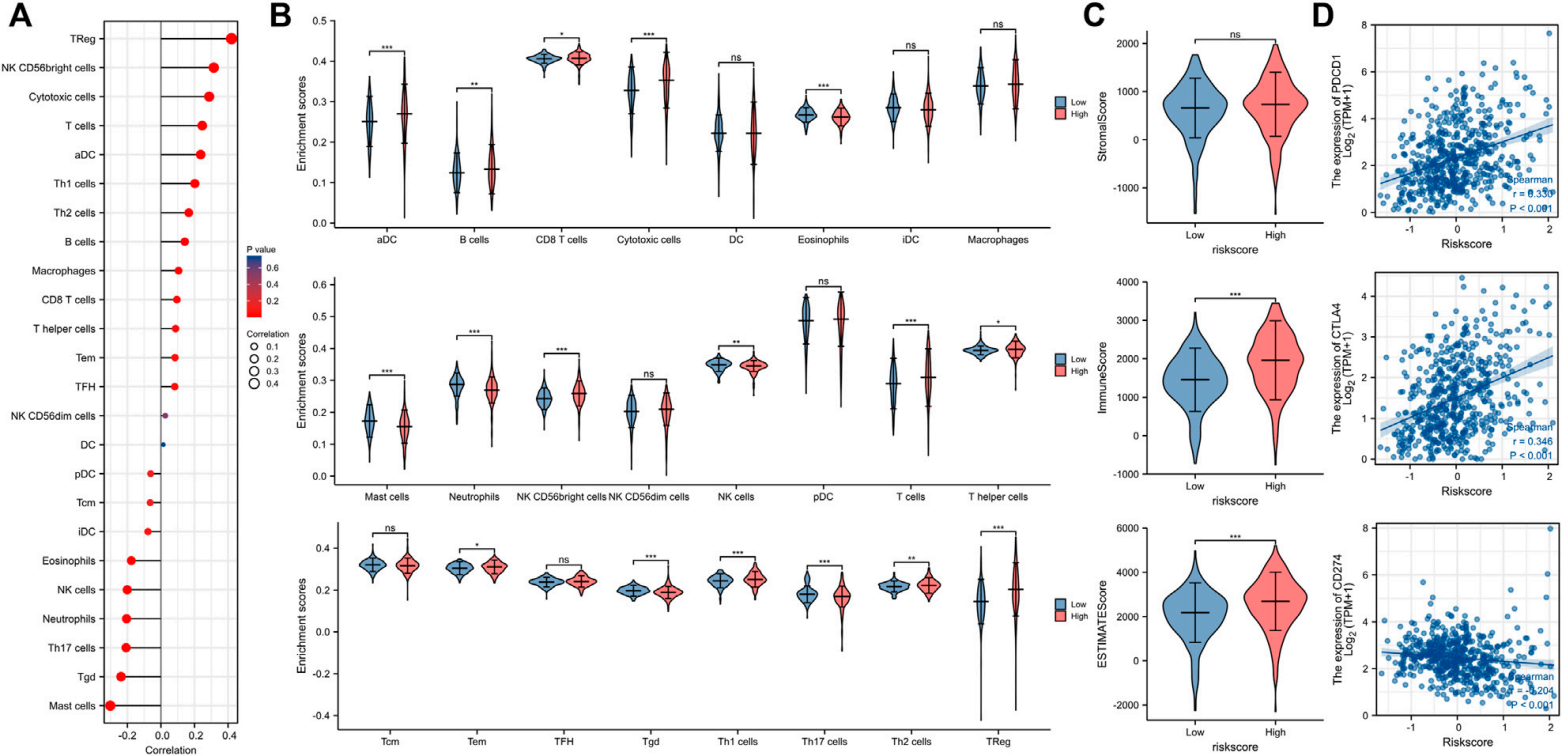
The tumor microenvironment and immune infiltration of ccRCC. **(A,B)** The infiltrating levels of 24 immune cell types between high-risk and low-risk groups. **(C)** The Stromal score, Immune score, and ESTIMATE score between high-risk and low-risk groups. **(D)** Relationship between Immune checkpoint molecules (PDCD1, CTLA4, CD274) and risk score. ccRCC, clear cell renal cell carcinoma; ns, *p* ≥ 0.05; *, *p* < 0.05; **, *p* < 0.01; ***, *p* < 0.001.

**FIGURE 8:**
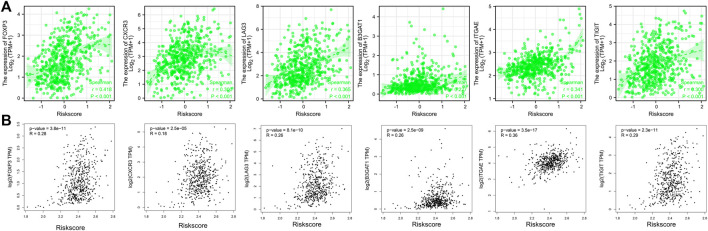
Correlation analysis between markers of Treg (FOXP3, CXCR3, LAG3, B3GAT1, ITGAE, TIGIT) and the risk score using TCGA **(A)** and GEPIA **(B)** database. TCGA, the cancer gene atlas; gepia, gene expression profiling interactive analysis.

We used the ESTIMATE and ssGSEA tools to evaluate disparities in tumor immune infiltration and immune cells between the high-risk and low-risk groups. Immune cell invasion was investigated using ssGSEA. Our results demonstrated large differences in the distribution of immunocytes between risk groups (Figures 7A,B). The immune infiltration of 24 kinds of immune cells for each TCGA sample can be found in Supplementary Table S6. Among numerous immunocytes, regulatory T cells (TRegs) were clearly increased in the high-risk group compared to the low-risk group. ImmuneScore and ESTIMATE Score in the high-risk group were also higher than those in the low-risk group (Figure 7C). Please see Supplementary Table S7 for more detailed information. Immune checkpoint molecules such as PDCD1, CTLA4, and CD274 were positively associated with the risk score (Figure 7D). Correlation analysis between markers of Treg (FOXP3, CXCR3, LAG3, B3GAT1, ITGAE, TIGIT) and the risk score using TCGA (Figure 8A) and GEPIA (Figure 8B) database further proved that Treg infiltration was positively related with the risk score.

### Establishment of normograms

**FIGURE 9:**
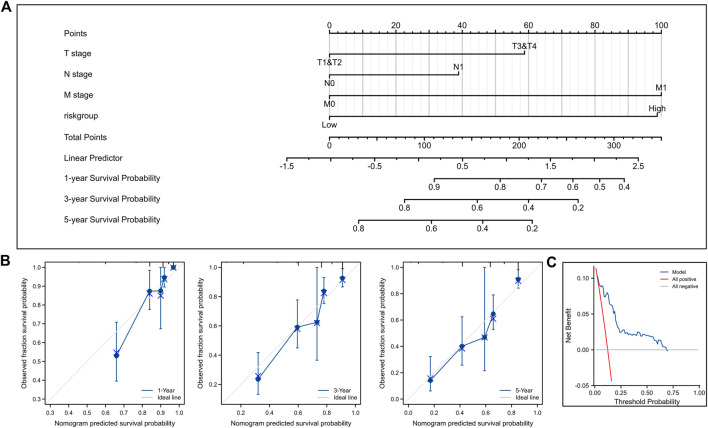
Construction and validation of nomogram for OS. **(A)** Nomogram to predict the 1-, 3-, and 5-year OS. **(B)** Calibration curves for the nomogram model. **(C)** Decision curve for the nomogram model. OS, overall survival.

We combined the risk score with clinical TNM stage of ccRCC to establish a Nomogram prognostic model for OS (Figure 9A). Calibration plots displayed good consistency between the predicted survival and the actual survival at 1, 3, and 5 years (Figure 9B). Decision curve also showed that the predictive power of our nomogram was satisfactory (Figure 9C).

**FIGURE 10:**
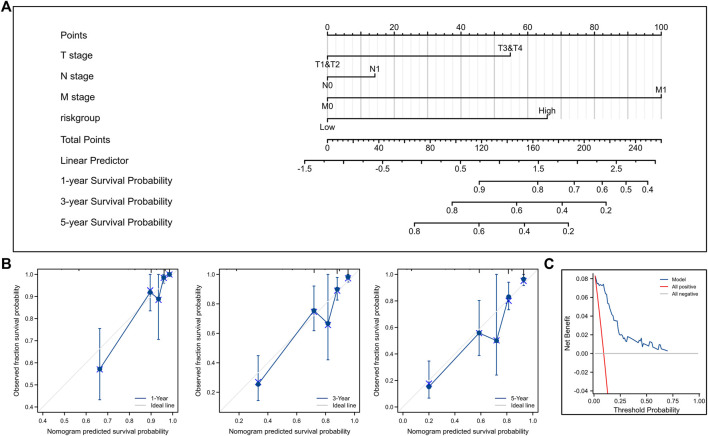
Construction and validation of nomogram for DSS. **(A)** Nomogram to predict the 1-, 3-, and 5-year DSS. **(B)** Calibration curves for the nomogram model. **(C)** Decision curve for the nomogram model. DSS, disease-specific survival.

Also, we established a nomogram for DSS (Figure 10A). Calibration plots (Figure 10B) and decision curve (Figure 10C) showed that the predictive ability of our nomogram was very good.

## Discussion

Radical or partial nephrectomy remains the mainstay of treatment for patients with primary ccRCC (Ljungberg et al., 2019). However, about 30% of patients will develop recurrence and metastasis after surgical treatment (Leibovich et al., 2018). Clinical salvage therapy approaches usually focus on the stage after tumor recurrence (Motzer et al., 2022). Therefore, there is an urgent need to establish an effective postoperative survival prediction model.

Previous studies showed that the pathogenesis of ccRCC was involved in multiple molecular pathways (Dizman et al., 2020; Zhang and Li, 2022; Yang, Ding, Sun, Rupprecht, Lin, Hsu, et al.). However, there are not many ccRCC prediction models involving multiple hotspot molecular pathways. We explored methylation, ferroptosis, pyroptosis, cuproptosis, and immune checkpoint genes associated with hub DEGs in ccRCC. Finally, the following five genes were included in the risk model: IGF2BP3, CDKN1A, GSDMB, FABP5, RBMX.

Among these five genes, IGF2BP3 and RBMX belong to the m6A signaling pathway. In previous studies of ccRCC, IGF2BP3 has been shown to be associated with poor tumor prognosis and metastasis (Jiang et al., 2008; Lederer et al., 2014). IGF2BP3 can interact with cell cycle kinases and extracellular matrix components to regulate the cell cycle (Gu et al., 2021). Moreover, Xie et al. (2021) concluded that IGF2BP3 was involved in the metabolism of IGF2BP3/CDKN2B-AS1/NUF2 axis, this genetic mechanism suggests that IGF2BP3 may be an ideal tumor marker and therapeutic target for ccRCC in the future. RBMX is a vital pathway molecule in the DNA damage repair mechanism (Adamson et al., 2012). Similarly, RBMX has been proved to inhibit the development of bladder cancer cells (Yan et al., 2021).

CDKN1A has been reported to be involved in ferroptosis. CDKN1A has also been shown to be associated with cystine metabolism in ccRCC (Yan et al., 2021). Knockdown of CDKN1A could promote melanoma proliferation in the G1 cell cycle (Jalili et al., 2012; Tarangelo et al., 2018). Also, Kramer et al. claimed that the LRH-1 gene suppressed the expression of CDKN1A, thereby driving colon cancer cell growth (Kramer et al., 2016).

GSDMB is associated with the pyroptosis pathway. Studies have shown that high expression of GSDMB can lead to the activation of the pyroptotic pathway in human monocyte cell lines, triggering non-apoptotic cell death (Chen et al., 2019). When the expression level of GSDMB is abnormally upregulated, the growth and invasive ability of bladder cancer can be significantly enhanced. GSDMB could bind to intracellular STAT3 and activate the STAT3 signaling pathway in bladder cancer (He et al., 2021). GSDMB has also been regarded as an effective biomarker of poor prognosis in ccRCC and a potential target for immunotherapy (Cui et al., 2021).

FABP5 is highly expressed in ccRCC compared to normal tissue and has prognostic significance based on multiple GEO gene chip analysis. Previous studies have shown that FABP5 may play an indispensable role in the angiogenesis of various tumors. FABP5 is highly upregulated in breast cancer and enhances proliferation, migration, and invasion. Artificial knockdown of FABP5 can significantly affect the activation of EGFR downstream genes (Levi et al., 2013). Also, FABP5 is critical in metastatic transformation and stromal cell interactions for triple-negative breast cancer (Apaya et al., 2020). FABP5 can promote epithelial-mesenchymal transition, lymphangiogenesis, and lymph node metastasis in cervical cancer cells by regulating fatty acid metabolism (Zhang et al., 2020b).

In summary, IGF2BP3, CDKN1A, GSDMB, FABP5, and RBMX genes are obviously correlated with the prognosis of ccRCC and could be used to establish a prognosis model. Patients in the low-risk group have significantly better survival compared with those in the high-risk group. Also, we found that risk score was strongly associated with survival status, tumor grade, and metastasis. We figured out that tissues with a high risk score tended to be prone to Treg infiltration, which predicted the onset of immune dysfunction (Díaz-Montero et al., 2020). The targeted drug sorafenib has been proved to reduce the number of immunosuppressive Tregs in ccRCC patients’ peripheral blood, thereby promoting the rebalancing of the immune environment in renal cancer (Desar et al., 2011). PD-1, CTLA4, and CD274 molecules are important immunotherapy targets in ccRCC, and their corresponding immune checkpoint inhibitors have been widely used in clinical treatment (Naranbhai et al., 2022; Navani and Heng, 2022). We demonstrated that our risk score was significantly correlated with the expression of immune checkpoint-related molecules. It can be seen that our model has a certain guiding effect on the immunotherapy of ccRCC.

After integrating our genetic prognostic risk model and clinical variables, we further developed a nomogram. The corresponding calibration and decision curve analysis showed that the performance of the nomogram was great.

However, several limitations should be noted in this study. The limitations of our study are the following: First, the outcome data were derived from public databases. Second, our study was a retrospective study, and patient sample volumes were limited. More real clinical data are needed to validate our findings. In the future, the expression condition of relevant key genes and their correlation with clinical stage could be verified in renal cancer cell lines and pathological tissues. Investigators can validate our derived prognostic model against the actual prognosis of the patients.

## Conclusion

We constructed a stratified model for ccRCC in this study. In terms of prognosis, high-risk patients have shorter OS than lower-risk patients. Early identification of such patients and effective intervention might help improve their long-term survival. The stratified model we constructed will help clinicians predict the prognosis of ccRCC and provide a good reference for clinical individualized treatment and immunotherapy options.

## Data Availability

The datasets presented in this study can be found in online repositories. The names of the repository/repositories and accession numbers can be found in the article/Supplementary Material.
